# Mean heart dose-based normal tissue complication probability model for pericardial effusion: a study in oesophageal cancer patients

**DOI:** 10.1038/s41598-021-97605-9

**Published:** 2021-09-13

**Authors:** Junichi Fukada, Kyohei Fukata, Naoyoshi Koike, Ryuichi Kota, Naoyuki Shigematsu

**Affiliations:** 1 Department of Radiology, Keio University School of Medicine, 35 Shinanomachi, Shinjuku-ku, Tokyo, 160-8582 Japan; 2 Cancer Center, Keio University School of Medicine, 35 Shinanomachi, Shinjuku-ku, Tokyo, 160-8582 Japan

**Keywords:** Risk factors, Cancer

## Abstract

We investigated the normal tissue complication probability (NTCP) of the incidence of pericardial effusion (PCE) based on the mean heart dose (MHD) in patients with oesophageal cancer treated with definitive chemoradiotherapy. The incidences of PCE in any grade (A-PCE) and symptomatic PCE (S-PCE) were evaluated separately. To identify predictors for PCE, several clinical and dose-volume parameters were analysed using a receiver operating characteristic (ROC) curve and multivariate regression analysis. To validate its clinical applicability, the generated NTCP model was compared to the Lyman–Kutcher–Burman (LKB) model. Among 229 eligible patients, A-PCE and S-PCE were observed in 100 (43.7%) and 18 (7.9%) patients, respectively. MHD showed a preferable area under the curve (AUC) value for S-PCE (AUC = 0.821) and A-PCE (AUC = 0.734). MHD was the only significant predictor for A-PCE; MHD and hypertension were selected as significant factors for S-PCE. The estimated NTCP, using the MHD-based model, showed excellent correspondence to the LKB model in A-PCE and S-PCE. The NTCP curve of A-PCE was gentler than that of S-PCE and had no threshold. The MHD-based NTCP model was simple but comparable to the LKB model for both A-PCE and S-PCE. Therefore, the estimated NTCP may provide clinically useful parameters for predicting PCE.

## Introduction

Radiation therapy plays an essential role in cancer treatment; however, cardiac toxicities are late adverse events associated with thoracic irradiation^1–11^. Partial volume radiation-induced heart complications mostly occur in long-surviving breast cancer patients who have undergone surgery^1,2^; meanwhile, evidence on radiation-related injuries after whole heart radiation has come mostly from patients with Hodgkin lymphoma^3^. Recently, cardiotoxicity has been recognised as an adverse event that occurs earlier than previously thought, as indicated by studies on patients with lung and oesophageal cancer^4–8^. Furthermore, some reports have suggested that heart irradiation might even affect short-term survival^6–8^. Among the various cardiac complications such as pericarditis, pericardial effusion (PCE), myocardial infarction, angina, and arrhythmia, PCE is regarded as the most frequent and important toxicity^2,4,8–11^. Although it has been suggested that reduced irradiation dose and volume delivered to the heart may help manage the risk of complications, the quantitative evaluation of the risk for dose-volume irradiation to the heart remains unclear. Therefore, it is reasonable to introduce mathematical modelling to analyse the radiation-to-heart dose-volume relationship and predict the normal tissue complication probability (NTCP).

We have previously reported the incidence of symptomatic PCE (S-PCE) in oesophageal cancer patients, showing that the average irradiation dose to the heart (including the pericardium) was the most significant predictor of S-PCE^11^. As the pericardium appears to be an anatomically uniform structure, we have assumed it to be a parallel organ. This study aimed to investigate the NTCP of PCE using mathematical modelling and examine whether the mean heart dose (MHD) can be used as an indicator of NTCP in S-PCE and PCE in any grade (A-PCE).

## Methods

The patient dataset analysed in this study was acquired from radiation treatment records of The Department of Radiation Oncology, Keio University Hospital. This was a retrospective observational study; hence, we used the “opt-out” method to obtain informed consent from patients. The study was approved by the Keio University School of Medicine Ethics Committee (no. 20150137). Patient clinical information was anonymised to protect personal information. All investigations were conducted in accordance with the relevant guidelines and regulations.

Patients were eligible for inclusion in the present study if they met the following criteria: newly diagnosed primary oesophageal cancer patients who received definitive concurrent chemoradiotherapy (CCR) between 2001 and 2014, involvement of the thoracic oesophagus, treated with conventional 2.0 or 1.8 Gy fractionation, total irradiation dose of ≥ 50 Gy, computed tomography (CT) data were available for analysis of the dose-volume of the pericardium, ≥ 6 months follow-up, and pathologically confirmed malignant PCE. Two-dimensional treatment plans were reconstructed as three-dimensional plans using a treatment planning system without modification, the details of which have been previously reported^11^.

Follow-up chest-abdominal CT scans and gastrointestinal fibrescopy were performed every 3–6 months for 3 years, and half-yearly to annual examinations were performed after 3 years. Adverse events were graded according to the National Cancer Institute-Common Terminology Criteria for Adverse Events (NCI-CTCAE) version 4.0^12^. S-PCE was defined as an effusion of grade 3 or above. The survival and PCE- free periods were determined after the completion of CCR. Heart volume is defined as the volume enclosed by the pericardium including the heart^11^. In this study, however, the pericardial volume was defined separately as a ring structure consisting of 2 mm of both the outer and inner walls of the heart. The cardiac substructures, including the right and left atria and the right and left ventricles, were also manually contoured on each CT image, referring to the Atlases for Organs at Risk (OARs) in Thoracic Radiation Therapy^13^ and the heart atlas by Feng et al.^14^. All contours were reviewed and corrected by a single radiation oncologist (J.F.). Three-dimensional plans were generated in increments of 10 cGy, using a commercially available radiation treatment planning system (XiO^®^, Elekta AB, Stockholm, Sweden), and calculated with the superposition algorithm appropriate for use with heterogeneous tissues. Calculated dose distribution was acquired by exporting the Digital Imaging and Communications in Medicine in radiation therapy (DICOM RT) file.

### Statistical calculation

Univariate logistic regression analysis was calculated with a cut-off p-value < 0.10 to analyse the relationship between PCE and patient and treatment characteristics. Pearson correlations were used to test multicollinearity with an R-squared threshold > 0.70. A multivariable forward stepwise logistic regression model was fit to include all selected significant factors associated with PCE. Variable parameters were included in the final model once the model was significantly better using the likelihood ratio test (p < 0.05, two-sided).

To validate our model internally, all patient and treatment factors and selected dosimetric parameters for the cause of the toxicities were repeated in 1000 bootstrap samples. The optimism of the generated model was assessed by estimating the performance difference between each bootstrap and the original sample in line with the Transparent Reporting of a multivariable prediction model for Individual Prognosis or Diagnosis (TRIPOD) statement^15^. The area and the adjusted area under the receiver operating characteristic (ROC) curve were compared to quantitatively evaluate the predictive power of the analyses. To perform an external validation, a previously reported model^16^ was compared to our models.

To compare and validate the MHD-based NTCP model, we used probit regression and compared it to the Lyman–Kutcher–Burman (LKB) NTCP model^17^. Generalised equivalent uniform dose (gEUD)-based LKB NTCP parameters (n, m, and TD_50_) were estimated using the maximum likelihood method^18^. In the probit regression-using MHD-based NTCP model, the m-value and TD_50_ were estimated at n = 1, with the pericardium considered an organ with a significant volume effect. In this case, TD_50_ represented MHD, instead of gEUD.

NTCP parameter optimisation from the DICOM RT file was performed using an in-house application. The application was coded with Python, Pydicom, dicompyler-core, and scikit-learn^19–22^. Optimum parameters were determined using the gradient descent method.

The probability of pericarditis incidence was given by

1 $$\begin{array}{c}NTCP=\frac{1}{\sqrt{2\pi }}{\int }_{-\infty }^{t}\mathrm{exp}\left(-\frac{{x}^{2}}{2}\right)dx\end{array}$$

with

2 $$\begin{array}{c}t=\frac{\mathrm{gEUD}-{\mathrm{TD}}_{50}}{m\cdot {\mathrm{TD}}_{50}}\end{array}$$

3 $$\begin{array}{c}gEUD={\left(\sum_{i}{v}_{i}{D}_{i}^\frac{1}{n}\right)}^{n}\end{array}$$

where n, m, and TD_50_ are the parameters of the LKB model, including the dose-volume effect on the organ; m-value represents the steepness of the dose–response curve, n-value represents the volume effect (large volume effect for n close to one; small volume effect for n close to zero), and TD_50_ represents the dose that corresponds to the 50% probability of complications, given whole-organ irradiation. In the third equation, v_i_ and D_i_ denote fractional volume and dose per ith voxel, respectively.

The following equation was used to calculate the coefficient of determination:

4 $${R}^{2}=1-\sum_{i}{\left({y}_{i}-f\right)}^{2}/\sum_{i}{\left({y}_{i}-\overline{y }\right)}^{2}$$

Herein, f denotes the linear function calculated by regression and $\overline{y }$ is the arithmetic mean of a vertical axis in the figure.

Statistical analyses were performed in SPSS, version 26.0 (SPSS Inc. Chicago Illinois, USA).

## Results

### Patient background and incidence of pericardial effusion

A total of 297 consecutive oesophageal cancer patients were treated with CCR during the study period. Cases were excluded when lost to follow-up before 6 months (n = 30), death occurring within 6 months (n = 24), no CT data available for dose-volume analysis (n = 13), malignant PCE (n = 1). Ultimately, 229 cases were included in this study. The overall median follow-up period was 37 months (range, 6–178), and that for surviving patients (n = 131) was 48 months (range, 6–178). A-PCE was observed in 100 (43.7%) patients. The timing of PCE onset ranged from 2–75 months, with a median of 7 months. S-PCE developed in 18 (7.9%) patients; the onset was 4–108 months, with a median of 21 months. Other observed new cardiac events were coronary artery disease (n = 8), arrhythmia (n = 6), and heart failure (n = 7). Within that group, six, three, and five patients also developed PCE. Patient demographic and clinical characteristics are summarised in Table 1.

**Table 1:** Demographic and clinical characteristics of patients included in the study cohort.

Characteristic	n = 229	(%)
**Age (years)**		
Median [range]	67	[43–87]
**Sex**		
Male	196	(85.6)
Female	33	(14.4)
**WHO performance status**		
0	69	(30.1)
1	134	(58.5)
2	26	(11.4)
**Hypertension**		
Yes	69	(30.1)
No	160	(69.9)
**Smoking history**		
Yes	55	(24.0)
No	171	(74.7)
Unknown	3	(1.3)
**Use of alcohol**		
Yes	27	(11.8)
No	196	(85.6)
Unknown	6	(2.6)
**Diabetes mellitus**		
Yes	27	(11.8)
No	202	(88.2)
**Cardiovascular disease**		
Yes	28	(12.2)
No	201	(87.8)
**Main tumour location**		
Ut	55	(24.0)
Mt	114	(49.8)
Lt	60	(26.2)
**Clinical stage**		
I	78	(34.1)
II	43	(18.8)
III	83	(36.2)
IV	25	(10.9)
**Heart volume (mL)**		
Median [range]	715	361–1188
**Radiation dose (Gy)**		
60	146	(63.8)
< 60	83	(36.2)
**Treatment planning**		
2D-plan	83	(36.2)
3D-CRT	146	(63.8)
**CCR regimen**		
CDDP + 5-FU	210	(91.7)
5-FU	3	(1.3)
CDDP + TS-1	9	(3.9)
Docetaxel	7	(3.1)
**MHD (Gy)**		
Median [range]	32.2	0.46–56.9

2D-plan: two-dimensional treatment plan; 3D-CRT: three-dimensional conformal radiotherapy; 5-FU: 5-fluorouracil; CCR: concurrent chemoradiotherapy; CDDP: cisplatin; Lt: lower thoracic oesophagus; MHD: mean heart dose; Mt: middle thoracic oesophagus; TS-1: Tegafur/Gimeracil/Oteracil; Ut: upper thoracic oesophagus; WHO: World Health Organization.

### Significant predictor identification for PCE by logistic regression analysis

Alcohol use was associated with A-PCE, while hypertension was associated with S-PCE by univariable logistic regression analysis. Meanwhile, most cardiopulmonary dose parameters were significantly associated with both A-PCE and S-PCE. Pearson’s correlation analysis revealed that these cardiac dose-volume parameters were highly correlated in predicting A-PCE and S-PCE. Then we performed a ROC analysis of these parameters to specify the most relevant parameter for PCE. MHD was selected as the significant predictor with the largest area under the curve (AUC) value for S-PCE (AUC = 0.821). While for A-PCE, the AUC of MHD (AUC = 0.713) was smaller than that of heart V40 (AUC = 0.734), we thought MHD could be regarded as a representative parameter considering its clinical usefulness and preferable AUC values. The detailed results of univariable logistic regression analyses and ROC analyses were provided in supplementary data (suppl. Data Tables 1 to 4).

**Figure 1:**
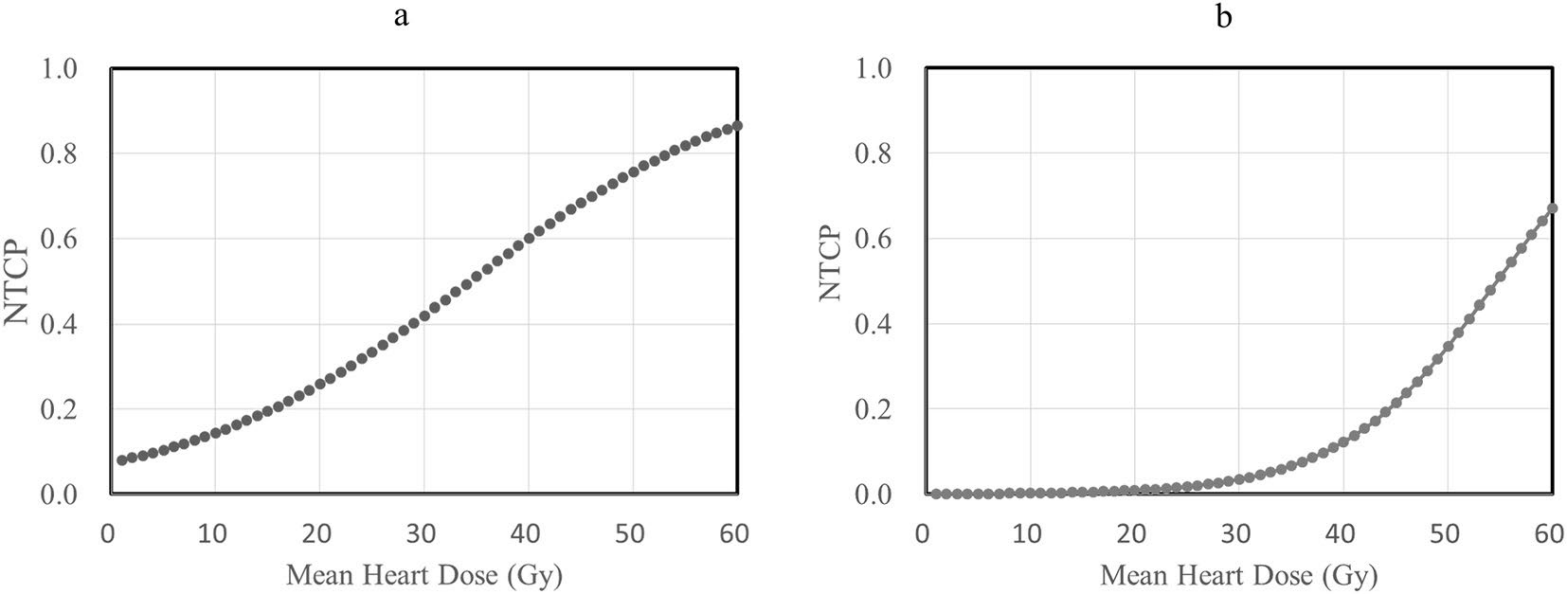
NTCP curves for A-PCE (**a**) and S-PCE (**b**). The NTCP curves were calculated by the logistic regression analysis derived from MHD. A-PCE, pericardial effusion in any grade; NTCP, normal tissue complicated probability; S-PCE, symptomatic pericardial effusion; MHD, mean heart dose.

**Figure 2:**
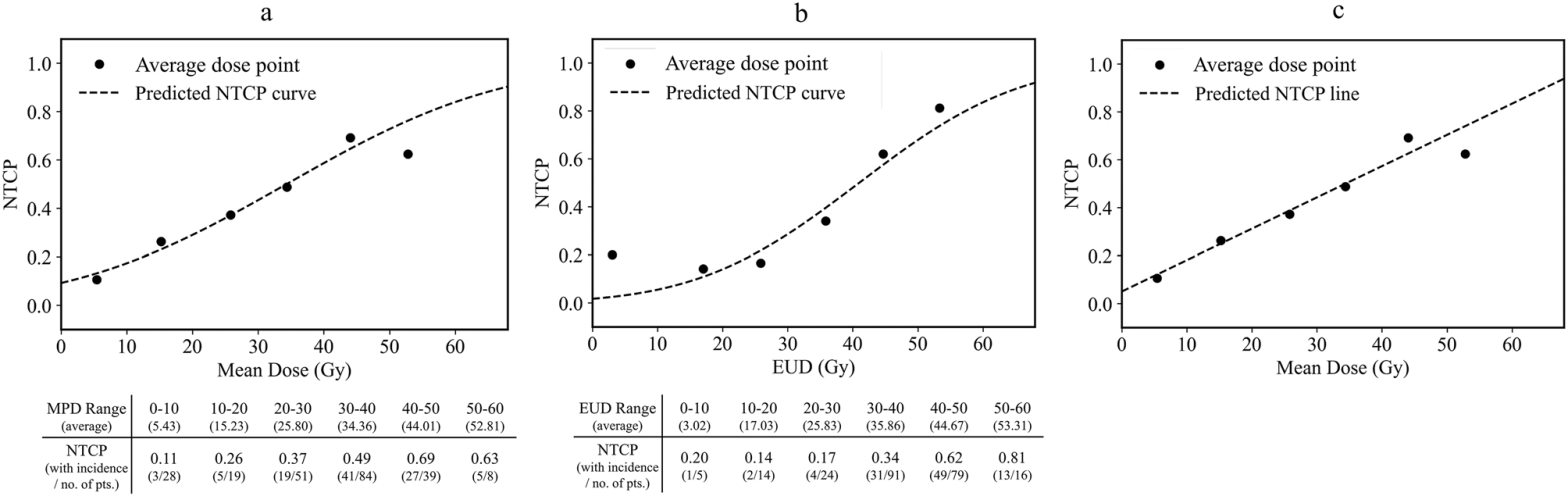
NTCP curves for A-PCE calculated by the MHD-based model (**a** and **c**) and LKB model (**b**). The x-axis indicates the MHD, which corresponds to the mean dose in the MHD-based model. Filled circles indicate average dose points for gEUD at 10 Gy intervals. NTCP curve by the MHD-based model also showed preferable fitting by linear regression (**c**). A-PCE, pericardial effusion in any grade; LKB model, Lyman–Kutcher–Burman model; MHD, mean heart dose; NTCP, normal tissue complicated probability; No. of Pts, number of patients; gEUD, generalised equivalent uniform dose.

**Figure 3:**
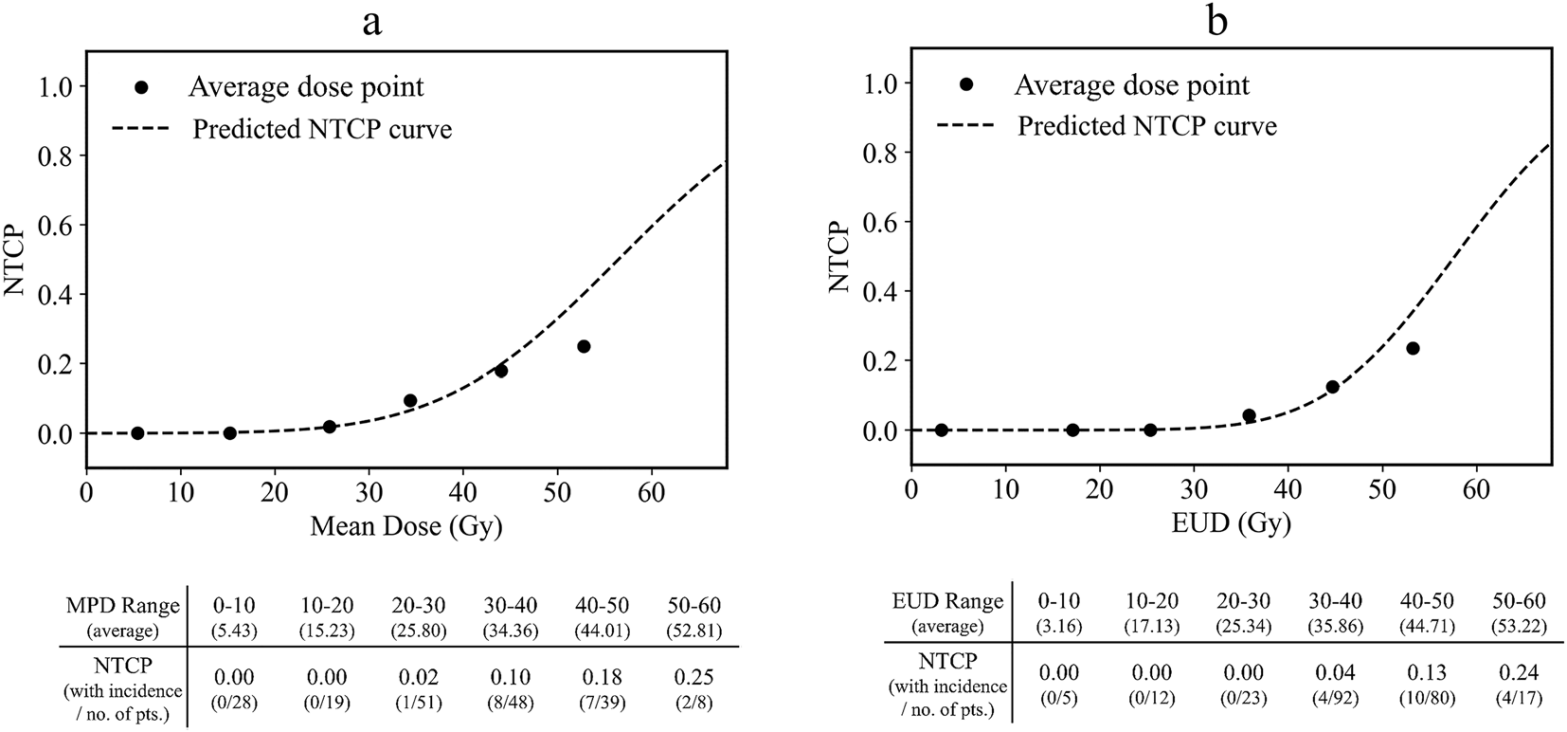
NTCP curves for S-PCE calculated by the (**a**) MHD-based model and (**b**) LKB model. The x-axis indicates gEUD, which corresponds to the mean dose in the MHD-based model. Filled circles indicate average dose points for gEUD at 10 Gy intervals. gEUD, generalised equivalent uniform dose; LKB model, Lyman–Kutcher–Burman model; No. of Pts, number of patients; NTCP, normal tissue complicated probability; S-PCE, symptomatic pericardial effusion; MHD, mean heart dose.

Multivariable logistic regression analysis demonstrated that only the MHD was the best predictor for A-PCE, with an odds ratio of 1.08 per Gy MHD (adjusted AUC after bootstrapping = 0.715). On the other hand, S-PCE was best predicted by MHD and hypertension, with an odds ratio of 1.17 (adjusted AUC after bootstrapping = 0.814). The estimated model and NTCP curve by logistic regression are presented in Table 2a and Fig. 1, respectively. The robustness of choosing MHD in the NTCP model, both for A-PCE and S-PCE, was verified by bootstrap analysis and calibration (suppl. Data Figs. 1 to 4). The previously reported NTCP model^16^ results are also shown in Table 2a as an external validation, indicating comparable results of discrimination (AUC) and calibration.

**Table 2:** NTCP models for A-PCE and S-PCE by logistic regression analyses (2a) and by probit regression analyses (2b).

(a)	Predictor	Coefficient	Constant	Odds ratio	CI (95%)	Significance	Discrimination (AUC)	Adjusted AUC	Hosmer–Lemeshow (chi-square, p)
A-PCE	MHD	0.073	− 2.513	1.076	1.05–1.11	0.000	0.713 [0.65–0.78]	0.715	13.05, 0.22
A-PCE^a^	MPD			1.11	1.06–1.16	0.00	0.73 [0.66–0.80]	0.70	2.86, 0.94
S-PCE	MHD	0.161	− 7.240	1.174	1.09–1.27	0.000	0.821 [0.73–0.91]	0.814	10.69, 0.36
	HTN	− 1.858		0.156	0.047–0.51	0.002			

(b)	Model	n	m	TD50	Log-likelihood	AUC	Hosmer–Lemeshow (chi-square, p)	Coefficient of determination	
A-PCE	LKB	0.38	0.47	41.0	− 140.90	0.718 [0.65–0.79]	8.55, 0.382	0.95	
	MHD-based	1	0.75	34.3	− 141.92	0.701 [0.63–0.77]	8.15, 0.418		
S-PCE	LKB	0.36	0.19	57.7	− 52.39	0.802 [0.68–0.93]	5.41, 0.713	0.95	
	MHD-based	1	0.26	56.5	− 51.94	0.809 [0.69–0.93]	8.64, 0.373		

A-PCE: pericardial effusion in any grade; AUC: area under the curve; CI (95%): 95% confidence interval; HTN: hypertension; LKB: Lyman–Kutcher–Burman; MHD: mean heart dose; MPD: mean pericardial dose; NTCP: normal tissue complication probability; S-PCE: symptomatic pericardial effusion; TD50: dose that corresponds to a 50% risk of complications when the whole organ is irradiated.

^a^A-PCE indicates pericardial effusion in any grade according to the NTCP model previously reported^16^.

### NTCP model by probit regression analysis

The NTCP curve for A-PCE derived from the MHD-based model calculated by probit regression and derived from the LKB model are shown in Fig. 2. The NTCP curve derived from the MHD-based model (Fig. 2a) showed a gentler curve (m = 0.75) than that derived from the LKB model (Fig. 2b) (m = 0.47); moreover, the NTCP curve derived from the MHD-based model showed preferable fitting by linear regression (Fig. 2c). The NTCP increased by 1.3% for each 1.0 Gy mean dose increase; the intercept was 5.1% at 0 Gy. When validating the goodness of fit, both the discrimination and the calibration of A-PCE in the MHD-based model were equivalent to that in the LKB model. On the other hand, the NTCP model for S-PCE derived from the MHD-based model calculated by probit regression and derived from the LKB model are shown in Fig. 3. The MHD-based model (Fig. 3a) was of similar shape and yielded comparable estimates as the LKB model (Fig. 3b). The validation of the goodness of fit, discrimination, and the calibration of S-PCE in the MHD-based model were equivalent to that in the LKB model. As a direct comparison of the MHD and LKB models, linear regression analysis and calibration results indicated excellent correspondence in both A-PCE and S-PCE. The summary of probit regression analysis was shown in Table 2b. The distribution of calibration plots was provided in supplementary data (suppl. data Figs. 5 to 8).

### Assessment of NTCP estimates

NTCP estimates derived from the logistic and probit regression are summarised in Table 3. The NTCP model for S-PCE showed a similar shape at the threshold near 30 Gy (Figs. 1b, 3a,b); however, the NTCP model for A-PCE, while also showing a similar shape, showed a curve that was gentler than that of S-PCE without threshold (Figs. 1a, 2a,b).

**Table 3:** Summary of the estimated TD and normal tissue complication probability stratified to the applied model and the grade of pericardial effusion.

	MHD-based (logistic)		MHD-based (probit)		LKB (probit)	
	A-PCE	S-PCE	A-PCE	S-PCE	A-PCE	S-PCE
**TD (Gy)**						
5%	–^a^	32.7	–^a^	32.4	9.0	39.9
10%	4.3	38.3	1.1	37.7	16.1	43.8
25%	19.4	46.5	16.9	46.6	27.9	50.4
50%	34.4	54.7	34.3	56.5	41.0	57.7
**NTCP (probability)**						
10 Gy	0.14	0.00	0.17	0.00	0.06	0.00
20 Gy	0.26	0.01	0.29	0.01	0.14	0.00
30 Gy	0.42	0.04	0.43	0.04	0.29	0.01
40 Gy	0.60	0.12	0.59	0.13	0.48	0.05
50 Gy	0.76	0.35	0.73	0.33	0.68	0.24

The upper row presents estimated TD_5_, _10_, _25_ and TD_50_ according to the calculated model and grade of PCE.

The bottom row presents the NTCP for each 10 Gy.

Regarding the LKB model, 10 Gy means gEUD whereas for the MHD-based model, 10 Gy means MHD. ^a^TD_5_ for the MHD-based model was not achieved because it was outside the fitting curve.

A-PCE: pericardial effusion in any grade; gEUD: generalised equivalent uniform dose; LKB: Lyman–Kutcher–Burman; MHD: mean heart dose; MPD-based: mean pericardial dose-based; NTCP: normal tissue complication probability; S-PCE: symptomatic pericardial effusion; TD: TD_50_ is the dose that corresponds to a 50% risk of complications when the whole organ is irradiated.

## Discussion

In this study, we investigated the NTCP of PCE using mathematical modelling. MHD was identified as the most significant predictor for S-PCE and a relevant parameter for A-PCE in multivariate analysis. The bootstrap analysis confirmed the robustness of obtaining a preferable AUC by selecting MHD in the NTCP model. The NTCP model derived from MHD was comparable to the widely used LKB model for both A-PCE and S-PCE.

PCE in cancer patients may arise through one of several mechanisms^23,24^. The lung disease study^10^ included receipt of adjuvant chemotherapy, history of cardiac disease, and a left-sided tumour, which were identified as independent predictors in the prospective subgroup. Several previous studies could not identify clinical background as a predictor for PCE^4,11,16^. In the current study, hypertension was selected as the only clinical factor relevant for S-PCE. A history of hypertension may induce cardiac symptoms in patients with A-PCE by exacerbating radiation-induced microvascular damage, raising jugular venous pressure, and edema. Chemotherapy may worsen the radiation-induced cardiotoxicity^23^, but it is challenging to elucidate the contribution of each therapy when CCR was performed as radical therapy. In our earlier study, chemotherapy was identified as a risk factor for PCE by univariate analysis but was not identified in the multivariate analysis^25^. Further investigation is needed to elucidate the mechanisms since PCE arises from various processes and factors.

In this study, various dose-volume parameters such as heart, pericardium, atria, and ventricles were widely selected as significant factors. These heart and cardiac substructures are highly correlated; therefore, we selected MHD as a representative of cardiac dose-volume parameters considering the results of ROC and the widely used guidelines^26^. Several previous studies have reported that the dose-volume of the irradiated pericardium may be a better factor than the dose volume of the whole heart^4,16^, and the bootstrap analysis of our study suggests the same. Since the dose volume of the whole heart was equivalent to that of the pericardium, we applied MHD to the NTCP model after considering its simplicity in clinical application. Some previous studies have investigated NTCP of cardiac toxicities in oesophageal cancer patients. According to studies by Emami and Burman et al., which proposed the LKB model, TD_50_ for the development of pericarditis was 48 Gy, with n- and m-value of 0.35 and 0.10, respectively^17,27^. In the present study, the estimated TD_50_ and n-values corresponding to S-PCE onset were nearly equivalent to those reported previously^17,27^. Therefore, the dose-volume associated with the development of pericarditis may correspond to that related to the development of S-PCE.

Meanwhile, several studies had investigated NTCP for A-PCE. Martel et al. reported an n-value of 0.63 for PCE, indicating that the pericardium is a parallel organ. These authors evaluated TD_50_ as a ‘biodose’, based on the data from a prospective clinical trial using a hypofractionation schedule, showing that TD_50_ was 50.6 Gy and m-value was 0.13, both of which are estimates comparable to those reported in the present study^28^. Recently, Beukema et al. reported NTCP for A-PCE using pericardial mean dose by logistic analysis^16^. Results of our external validation were per the TRIPOD statement, as the discrimination ability and calibration were comparable to their results. In this study, the NTCP curve for A-PCE showed a gentler shape than S-PCE without threshold. In addition, A-PCE showed preferable fitting by a straight line with intercept, indicating that the risk of A-PCE increases linearly with MHD. A previous report on the development of coronary artery disease in breast cancer patients revealed that disease risk increased linearly with the administration of MHD^29^. Similarly, another breast cancer study reported a predication model for acute cardiac events that concluded that LV-V5 (left ventricular volume receiving 5 Gy) was the predictor^30^. These results may suggest that even low doses cause cardiotoxicity.

Some previous studies have assumed n to equal one when considering other organs at risk to be parallel organs, for example, lungs, salivary glands, and liver^31–33^. For example, a previous study of radiation pneumonitis by Semenenko and Li compared NTCP estimates of cases with steroid administration with those of all instances showing that the NTCP sigmoid curve for all events followed a gentler progression with a lower threshold of mean total lung dose than that for cases of severe pneumonitis that required treatment with steroids^32^. These findings correspond to the present findings on NTCP curves for A-PCE and S-PCE.

This study has some limitations, which should be considered when interpreting its findings. First, as this was a retrospective single-centre study, the sample size and the number of symptomatic cardiac events except for PCE was small. Hence, the estimated NTCP was limited to PCE, not to whole cardiac events. Second, the estimated dose volume of cases treated with a two-dimensional plan might be inaccurate due to reconstruction. Since S-PCE was observed relatively frequently in the early period, we included these initial cases in the NTCP analysis. External validation using studies performed on modern irradiation techniques, such as intensity moderated radiation therapy^34^, may strengthen our model. Third, checking the inter-operator consistency with two oncologists would be beneficial since definition of border of pericardium is not easy. Noticeably, mathematical models have inherent limitations, as they do not consider host factors.

These limitations notwithstanding, the present study has several strengths. A substantial number of cases were treated using conventional fractionated irradiation and cardiac and lung structures were delineated by methods that have been previously reported as atlases and are now included among the standard. We clarify the difference in the NTCP curve for A-PCE and S-PCE. The risk of PCE could be predicted from the MHD, and it can be easily referred in daily radiotherapy planning. We believe that it can contribute to safe radiotherapy. Although the reported n-value was consistent with that shown in a previous study, we could not demonstrate that the pericardium was a parallel organ. Further studies may elucidate the impact of irradiation on a specific cardiac substructure encompassed by the pericardium.

## Conclusions

The MHD was identified as a significant predictor for PCE risk assessment with preferable ROC and robustness. The MHD-based NTCP model is simple but comparable to the LKB model for both A-PCE and S-PCE. Estimated NTCP provides clinically useful parameters for predicting PCE. Hypertension may accelerate symptoms in the presence of PCE.

## Supplementary Information

Supplementary Information.
