# Supplementary material for: Mean heart dose-based normal tissue complication probability model for pericardial effusion: a study in oesophageal cancer patients
Source: Sci Rep. 2021 Sep 13;11:18166. doi: 10.1038/s41598-021-97605-9 (PMC8437977; doi:10.1038/s41598-021-97605-9)
Supplement: Supplementary file 1 — Supplementary Information. [file 41598_2021_97605_MOESM1_ESM.pdf]

Mean heart dose-based normal tissue complication probability model for pericardial effusion —a study in oesophageal cancer patients

Junichi Fukada*[a], Kyohei Fukata[a,b], Naoyoshi Koike[a], Ryuichi Kota[a], Naoyuki Shigematsu[a]

[a]Department of Radiology, School of Medicine, Keio University, School of Medicine, 35 Shinanomachi, Shinjuku-ku, Tokyo 160-8582, Japan

[b]Cancer Center, School of Medicine, Keio University, School of Medicine, 35 Shinanomachi, Shinjuku-ku, Tokyo 160-8582, Japan

***Corresponding author:**

Junichi Fukada

Department of Radiology, Keio University, School of Medicine, 35 Shinanomachi, Shinjuku-ku, Tokyo 160-8582, Japan

Tel: +81-3-3353-1211

Fax: +81-3-3359-7425

E-mail: fukada@rad.med.keio.ac.jp

Supplementary Files

Supplementary Table 1. Univariate logistic regression analyses for A-PCE

	Score	Degree	p-value
**Age**	0.648	1	0.421
**Sex (male=0, female=1)**	1.447	1	0.229
**Hypertension (1)**	1.696	1	0.193
**Cardiac disease history (1)**	0.386	1	0.535
**Smoking (1)**	0.389	1	0.533
**Diabetes mellitus (1)**	1.124	1	0.289
**Alcohol (1)**	3.542	1	0.060
**PS**	0.451	2	0.798
**PS (1)**	0.176	1	0.675
**PS (2)**	0.429	1	0.512
**Pericardium volume**	2.135	1	0.144
**Clinical stage (I vs. II-IV)**	0.944	1	0.331
**2D-plan/3D-CRT**	4.969	1	0.026
**Radiation dose 60 Gy (1)**	3.599	1	0.058
**Heart average dose**	31.293	1	0.000
**Heart V10**	19.621	1	0.000
**Heart V20**	24.251	1	0.000
**Heart V30**	27.368	1	0.000
**Heart V40**	37.236	1	0.000
**Heart V50**	33.776	1	0.000
**Heart V60**	16.272	1	0.000
**Pericardium average dose**	32.788	1	0.000
**Pericardium V10**	24.019	1	0.000
**Pericardium V20**	30.026	1	0.000
**Pericardium V30**	30.812	1	0.000
**Pericardium V40**	34.595	1	0.000
**Pericardium V50**	28.748	1	0.000
**Pericardium V60**	13.259	1	0.000
**Lt Atrium average dose**	23.854	1	0.000
**Lt Atrium V10**	16.615	1	0.000
**Lt Atrium V20**	18.129	1	0.000

**Lt Atrium V30**	19.252	1	0.000
**Lt Atrium V40**	24.291	1	0.000
**Lt Atrium V50**	26.266	1	0.000
**Lt Atrium V60**	5.312	1	0.021
**Rt Atrium average dose**	25.347	1	0.000
**Rt Atrium V10**	17.167	1	0.000
**Rt Atrium V20**	18.871	1	0.000
**Rt Atrium V30**	19.507	1	0.000
**Rt Atrium V40**	25.216	1	0.000
**Rt Atrium V50**	20.61	1	0.000
**Rt Atrium V60**	9.720	1	0.002
**Lt Ventricle average dose**	12.244	1	0.000
**Lt Ventricle V10**	9.048	1	0.003
**Lt Ventricle V20**	11.677	1	0.001
**Lt Ventricle V30**	12.337	1	0.000
**Lt Ventricle V40**	13.462	1	0.000
**Lt Ventricle V50**	9.488	1	0.002
**Lt Ventricle V60**	1.808	1	0.179
**Rt Ventricle average dose**	20.667	1	0.000
**Rt Ventricle V10**	13.567	1	0.000
**Rt Ventricle V20**	14.871	1	0.000
**Rt Ventricle V30**	16.272	1	0.000
**Rt Ventricle V40**	27.984	1	0.000
**Rt Ventricle V50**	12.364	1	0.000
**Rt Ventricle V60**	4.965	1	0.026
**Lt Lung average dose**	8.498	1	0.004
**Lt Lung V10**	5.034	1	0.025
**Lt Lung V20**	4.919	1	0.027
**Lt Lung V30**	5.467	1	0.019
**Lt Lung V40**	7.155	1	0.007
**Lt Lung V50**	6.464	1	0.011
**Lt Lung V60**	4.320	1	0.038
**Rt Lung average dose**	6.641	1	0.010
**Rt Lung V10**	3.342	1	0.068
**Rt Lung V20**	7.048	1	0.008
**Rt Lung V30**	4.391	1	0.036

**Rt Lung V40**	4.733	1	0.030
**Rt Lung V50**	2.507	1	0.113
**Rt Lung V60**	3.023	1	0.082
	96.584	69	0.016

2D-plan: two-dimensional treatment plan; 3D-CRT: three-dimensional conformal radiotherapy; A-PCE: pericardial effusion of any grade; Degree: degree of freedom; Lt: left; PS: performance state; Rt: right.

Supplementary Table 2. Univariate logistic regression analyses for S-PCE

	Score	Degree	p-value
**Age**	2.762	1	0.097
**Sex (male=0, female=1)**	1.246	1	0.264
**Hypertension (1)**	5.986	1	0.014
**Cardiac disease history (1)**	0.007	1	0.934
**Smoking (1)**	1.655	1	0.198
**Diabetes mellitus (1)**	2.093	1	0.148
**Alcohol (1)**	2.598	1	0.107
**PS**	0.631	2	0.729
**PS (1)**	0.589	1	0.443
**PS (2)**	0.531	1	0.466
**Pericardium volume**	0.000	1	0.998
**Clinical stage (I vs. II-IV)**	2.932	1	0.087
**2D-plan/3D-CRT**	9.250	1	0.002
**Radiation dose 60 Gy (1)**	3.320	1	0.068
**Heart average dose**	18.847	1	0.000
**Heart V10**	11.858	1	0.001
**Heart V20**	13.555	1	0.000
**Heart V30**	17.217	1	0.000
**Heart V40**	20.217	1	0.000
**Heart V50**	18.691	1	0.000
**Heart V60**	15.472	1	0.000
**Pericardium average dose**	20.387	1	0.000
**Pericardium V10**	13.437	1	0.000
**Pericardium V20**	15.293	1	0.000
**Pericardium V30**	19.598	1	0.000
**Pericardium V40**	19.429	1	0.000
**Pericardium V50**	23.143	1	0.000
**Pericardium V60**	15.094	1	0.000
**Lt Atrium average dose**	9.082	1	0.003
**Lt Atrium V10**	4.910	1	0.027
**Lt Atrium V20**	5.533	1	0.019
**Lt Atrium V30**	6.770	1	0.009

**Lt Atrium V40**	8.998	1	0.003
**Lt Atrium V50**	13.191	1	0.000
**Lt Atrium V60**	1.717	1	0.190
**Rt Atrium average dose**	11.55	1	0.001
**Rt Atrium V10**	7.255	1	0.007
**Rt Atrium V20**	5.334	1	0.021
**Rt Atrium V30**	8.800	1	0.003
**Rt Atrium V40**	11.056	1	0.001
**Rt Atrium V50**	15.350	1	0.000
**Rt Atrium V60**	7.517	1	0.006
**Lt Ventricle average dose**	13.178	1	0.000
**Lt Ventricle V10**	11.089	1	0.001
**Lt Ventricle V20**	12.640	1	0.000
**Lt Ventricle V30**	13.239	1	0.000
**Lt Ventricle V40**	14.807	1	0.000
**Lt Ventricle V50**	4.887	1	0.027
**Lt Ventricle V60**	4.079	1	0.043
**Rt Ventricle average dose**	13.022	1	0.000
**Rt Ventricle V10**	7.937	1	0.005
**Rt Ventricle V20**	9.369	1	0.002
**Rt Ventricle V30**	11.416	1	0.001
**Rt Ventricle V40**	16.701	1	0.000
**Rt Ventricle V50**	5.943	1	0.015
**Rt Ventricle V60**	4.108	1	0.043
**Lt Lung average dose**	10.265	1	0.001
**Lt Lung V10**	4.893	1	0.027
**Lt Lung V20**	7.342	1	0.007
**Lt Lung V30**	9.299	1	0.002
**Lt Lung V40**	10.195	1	0.001
**Lt Lung V50**	14.653	1	0.000
**Lt Lung V60**	15.853	1	0.000
**Rt Lung average dose**	6.674	1	0.010
**Rt Lung V10**	4.940	1	0.026
**Rt Lung V20**	5.341	1	0.021
**Rt Lung V30**	4.883	1	0.027
**Rt Lung V40**	4.088	1	0.043

**Rt Lung V50**	7.144	1	0.008
**Rt Lung V60**	1.958	1	0.162
	93.881	69	0.025

2D-plan: two-dimensional treatment plan; 3D-CRT: three-dimensional conformal radiotherapy; Degree: degree of freedom; Lt: left; PS: performance status; Rt: right; S-PCE: symptomatic pericardial effusion.

Supplementary Table 3. ROC analyses for A-PCE

Variables	AUC	p-value	95% CI
**Heart average dose**	0.713	0.000	0.647-0.779
**Heart V10**	0.660	0.000	0.589-0.730
**Heart V20**	0.684	0.000	0.616-0.753
**Heart V30**	0.696	0.000	0.629-0.764
**Heart V40**	0.734	0.000	0.669-0.798
**Heart V50**	0.732	0.000	0.667-0.797
**Heart V60**	0.637	0.000	0.564-0.711
**Pericardium average dose**	0.717	0.000	0.651-0.784
**Pericardium V10**	0.689	0.000	0.621-0.757
**Pericardium V20**	0.708	0.000	0.641-0.775
**Pericardium V30**	0.709	0.000	0.642-0.776
**Pericardium V40**	0.725	0.000	0.659-0.790
**Pericardium V50**	0.717	0.000	0.650-0.784
**Pericardium V60**	0.628	0.001	0.555-0.702
**Lt Atrium average dose**	0.689	0.000	0.621-0.758
**Lt Atrium V10**	0.633	0.000	0.562-0.705
**Lt Atrium V20**	0.667	0.000	0.597-0.737
**Lt Atrium V30**	0.675	0.000	0.605-0.744
**Lt Atrium V40**	0.697	0.000	0.629-0.764
**Lt Atrium V50**	0.691	0.000	0.623-0.760
**Lt Atrium V60**	0.546	0.231	0.470-0.622
**Rt Atrium average dose**	0.688	0.000	0.620-0.757
**Rt Atrium V10**	0.664	0.000	0.594-0.734
**Rt Atrium V20**	0.673	0.000	0.603-0.742
**Rt Atrium V30**	0.678	0.000	0.608-0.747
**Rt Atrium V40**	0.692	0.000	0.624-0.761
**Rt Atrium V50**	0.665	0.000	0.593-0.737
**Rt Atrium V60**	0.566	0.088	0.490-0.642
**Lt Ventricle average dose**	0.640	0.000	0.568-0.712
**Lt Ventricle V10**	0.622	0.001	0.549-0.694
**Lt Ventricle V20**	0.639	0.000	0.567-0.710

**Lt Ventricle V30**	0.643	0.000	0.571-0.715
**Lt Ventricle V40**	0.661	0.000	0.590-0.732
**Lt Ventricle V50**	0.640	0.000	0.566-0.713
**Lt Ventricle V60**	0.536	0.349	0.460-0.612
**Rt Ventricle average dose**	0.684	0.000	0.615-0.754
**Rt Ventricle V10**	0.645	0.000	0.573-0.716
**Rt Ventricle V20**	0.661	0.000	0.590-0.731
**Rt Ventricle V30**	0.668	0.000	0.598-0.738
**Rt Ventricle V40**	0.708	0.000	0.640-0.775
**Rt Ventricle V50**	0.650	0.000	0.578-0.723
**Rt Ventricle V60**	0.549	0.211	0.473-0.625
**Lt Lung average dose**	0.604	0.005	0.531-0.677
**Lt Lung V10**	0.589	0.018	0.515-0.663
**Lt Lung V20**	0.580	0.034	0.506-0.655
**Lt Lung V30**	0.586	0.024	0.512-0.660
**Lt Lung V40**	0.603	0.006	0.530-0.677
**Lt Lung V50**	0.591	0.016	0.517-0.666
**Lt Lung V60**	0.586	0.024	0.512-0.661
**Rt Lung average dose**	0.599	0.008	0.525-0.672
**Rt Lung V10**	0.564	0.090	0.490-0.639
**Rt Lung V20**	0.599	0.008	0.525-0.672
**Rt Lung V30**	0.592	0.014	0.518-0.665
**Rt Lung V40**	0.602	0.006	0.529-0.675
**Rt Lung V50**	0.598	0.010	0.524-0.672
**Rt Lung V60**	0.556	0.145	0.481-0.632

A-PCE: pericardial effusion of any grade; AUC: area under the curve; CI: confidence interval; Lt: left; ROC: receiver operating characteristic; Rt: right.

Supplementary Table 4. ROC analyses for S-PCE

Variables	AUC	p-value	95% CI
**Heart average dose**	0.821	0.000	0.733-0.909
**Heart V10**	0.797	0.000	0.707-0.886
**Heart V20**	0.793	0.000	0.701-0.884
**Heart V30**	0.819	0.000	0.734-0.904
**Heart V40**	0.817	0.000	0.740-0.894
**Heart V50**	0.776	0.000	0.679-0.874
**Heart V60**	0.685	0.013	0.539-0.831
**Pericardium average dose**	0.804	0.000	0.704-0.903
**Pericardium V10**	0.793	0.000	0.701-0.886
**Pericardium V20**	0.777	0.000	0.672-0.882
**Pericardium V30**	0.806	0.000	0.713-0.899
**Pericardium V40**	0.800	0.000	0.712-0.887
**Pericardium V50**	0.792	0.000	0.695-0.889
**Pericardium V60**	0.691	0.007	0.553-0.829
**Lt Atrium average dose**	0.764	0.000	0.659-0.869
**Lt Atrium V10**	0.691	0.000	0.594-0.789
**Lt Atrium V20**	0.715	0.000	0.610-0.821
**Lt Atrium V30**	0.750	0.000	0.650-0.851
**Lt Atrium V40**	0.764	0.000	0.670-0.859
**Lt Atrium V50**	0.760	0.000	0.650-0.870
**Lt Atrium V60**	0.573	0.322	0.429-0.717
**Rt Atrium average dose**	0.731	0.000	0.615-0.846
**Rt Atrium V10**	0.699	0.000	0.596-0.801
**Rt Atrium V20**	0.680	0.002	0.564-0.797
**Rt Atrium V30**	0.716	0.000	0.605-0.828
**Rt Atrium V40**	0.729	0.000	0.622-0.837
**Rt Atrium V50**	0.746	0.000	0.631-0.862
**Rt Atrium V60**	0.662	0.029	0.516-0.808
**Lt Ventricle average dose**	0.761	0.000	0.664-0.859
**Lt Ventricle V10**	0.742	0.000	0.642-0.843
**Lt Ventricle V20**	0.761	0.000	0.664-0.857

**Lt Ventricle V30**	0.768	0.000	0.673-0.863
**Lt Ventricle V40**	0.778	0.000	0.680-0.876
**Lt Ventricle V50**	0.687	0.002	0.566-0.808
**Lt Ventricle V60**	0.550	0.507	0.403-0.696
**Rt Ventricle average dose**	0.789	0.000	0.698-0.881
**Rt Ventricle V10**	0.712	0.000	0.615-0.809
**Rt Ventricle V20**	0.765	0.000	0.668-0.862
**Rt Ventricle V30**	0.793	0.000	0.694-0.892
**Rt Ventricle V40**	0.790	0.000	0.703-0.877
**Rt Ventricle V50**	0.705	0.001	0.582-0.829
**Rt Ventricle V60**	0.568	0.369	0.420-0.716
**Lt Lung average dose**	0.668	0.004	0.554-0.782
**Lt Lung V10**	0.637	0.028	0.515-0.758
**Lt Lung V20**	0.639	0.027	0.516-0.762
**Lt Lung V30**	0.650	0.019	0.525-0.775
**Lt Lung V40**	0.666	0.010	0.539-0.793
**Lt Lung V50**	0.693	0.002	0.572-0.815
**Lt Lung V60**	0.676	0.006	0.550-0.802
**Rt Lung average dose**	0.680	0.005	0.555-0.806
**Rt Lung V10**	0.668	0.008	0.543-0.793
**Rt Lung V20**	0.660	0.020	0.525-0.795
**Rt Lung V30**	0.659	0.011	0.537-0.781
**Rt Lung V40**	0.638	0.039	0.507-0.770
**Rt Lung V50**	0.696	0.002	0.569-0.824
**Rt Lung V60**	0.623	0.110	0.472-0.773

AUC: area under the curve; CI: confidence interval; Lt: left; ROC: receiver operating characteristic; Rt: right; S-PCE: symptomatic pericardial effusion.

Supplementary Figure 1. Selected frequency for A-PCE by bootstrap analysis

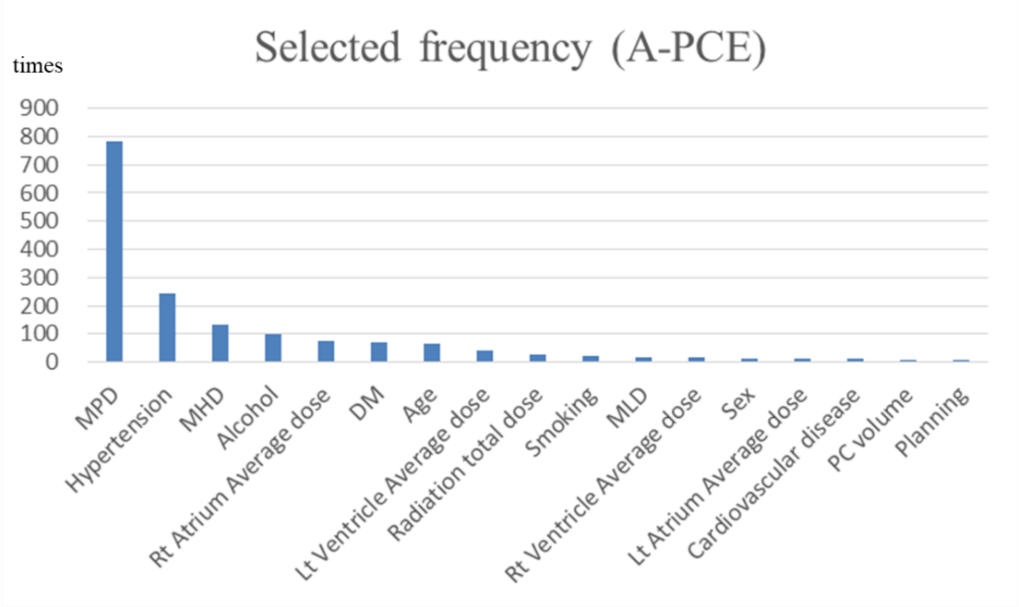

A-PCE: pericardial effusion in any grade; DM: diabetes mellitus; Lt: left; MHD: mean heart dose; MLD: mean lung dose; MPD: mean pericardial dose; PC: pericardium; PS: performance status; Rt: right.

Supplementary Figure 2. Calibration plots using the Hosmer–Lemeshow test of the observed vs. calculated risk for A-PCE

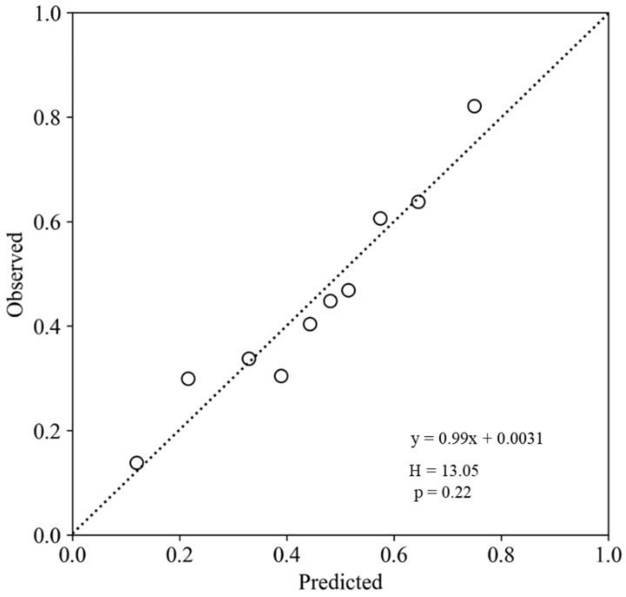

A-PCE: pericardial effusion in any grade

Supplementary Figure 3. Selected frequency for S-PCE by bootstrap analysis

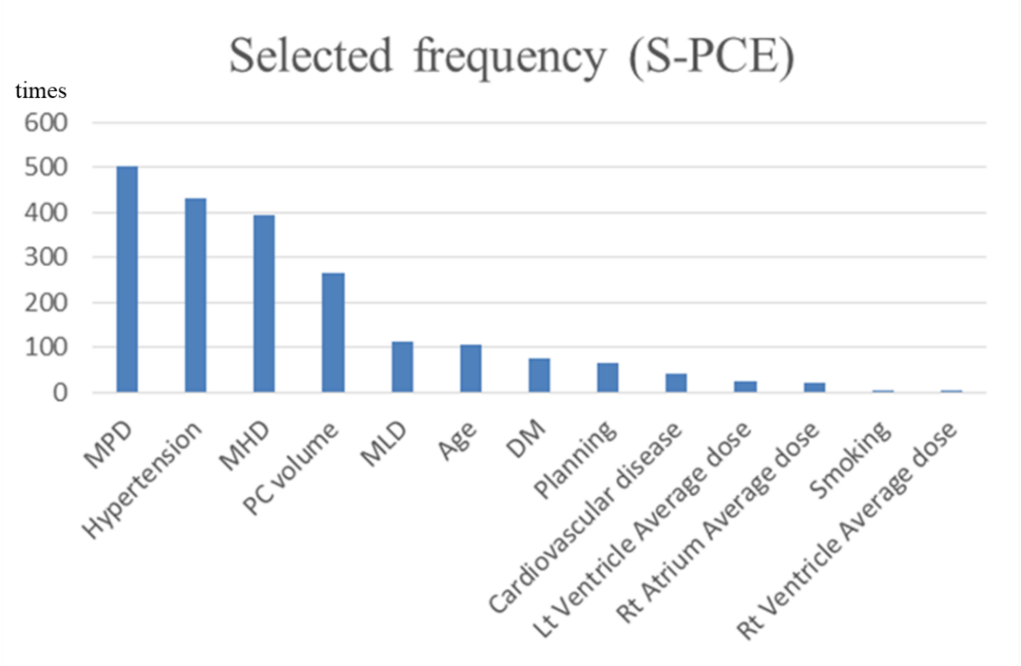

DM: diabetes mellitus; Lt: left; MHD: mean heart dose; MLD: mean lung dose; MPD: mean pericardial dose; PC: pericardium; Rt: right; S-PCE: symptomatic pericardial effusion.

Supplementary Figure 4. Calibration plots using the Hosmer–Lemeshow test of the observed vs. calculated risk for S-PCE

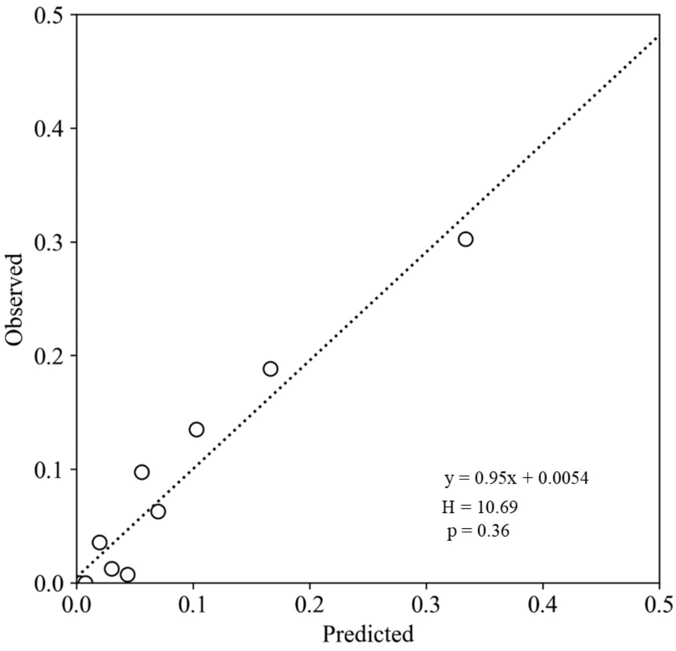

S-PCE: symptomatic pericardial effusion

Supplementary Figure 5. Calibration plots using the Hosmer–Lemeshow test of the observed vs. calculated risk by MHD (5a) and LKB (5b) model for A-PCE

(5a)

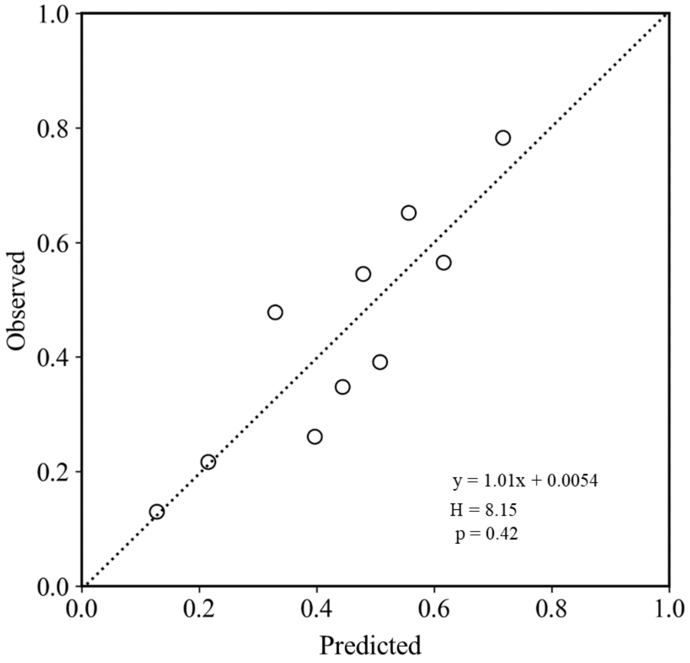

(5b)

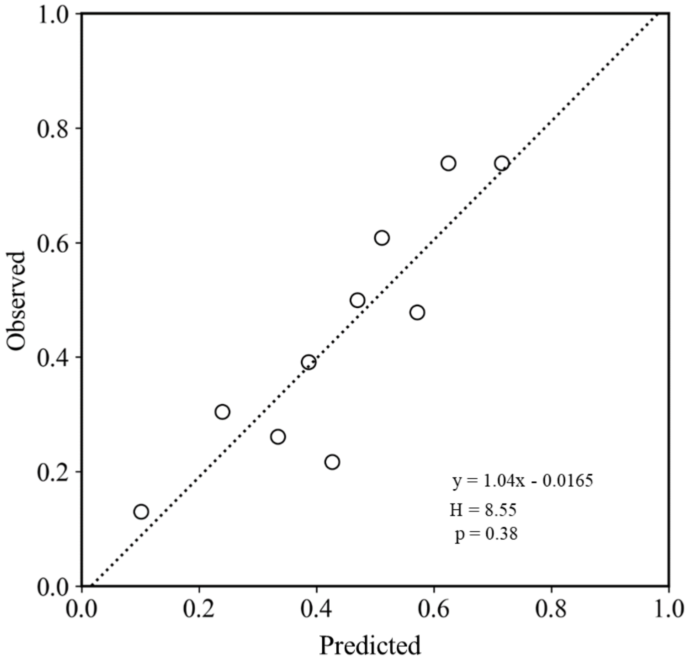

A-PCE: pericardial effusion in any grade; LKB: Lyman–Kutcher–Burman; MHD: mean heart dose

Supplementary Figure 6. Calibration plots using the Hosmer–Lemeshow test of the observed vs. calculated risk by MHD (6a) and LKB (6b) model for S-PCE

(6a)

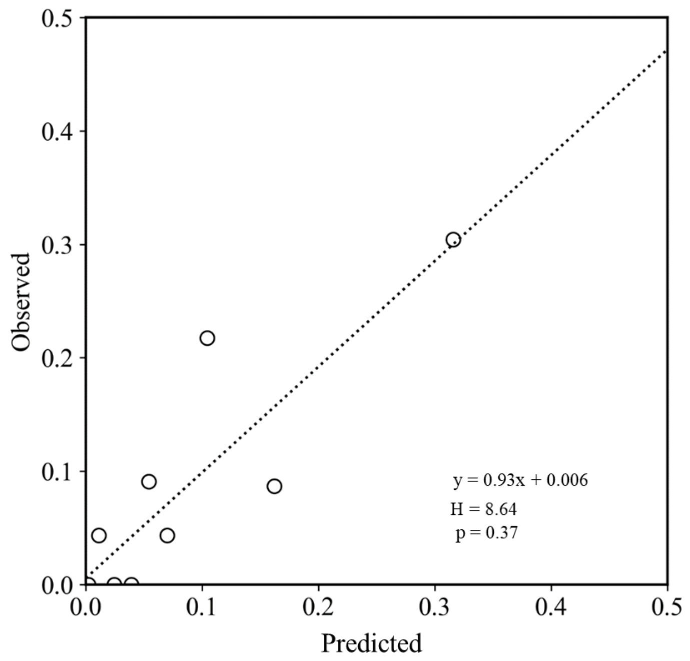

(6b)

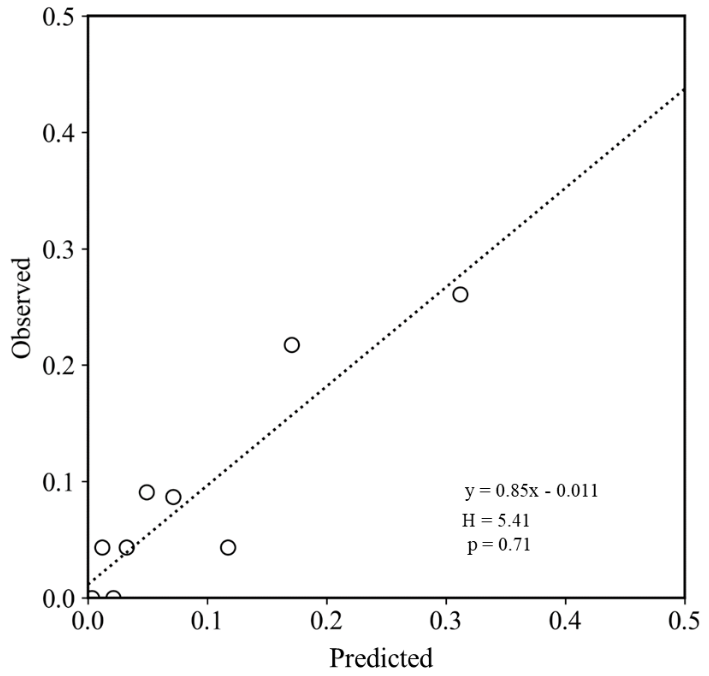

LKB: Lyman–Kutcher–Burman; MHD: mean heart dose; S-PCE: symptomatic pericardial effusion.

Supplementary Figure 7. Calibration plots using the Hosmer–Lemeshow test of the predicted risk by MHD-based model vs. LKB model for A-PCE

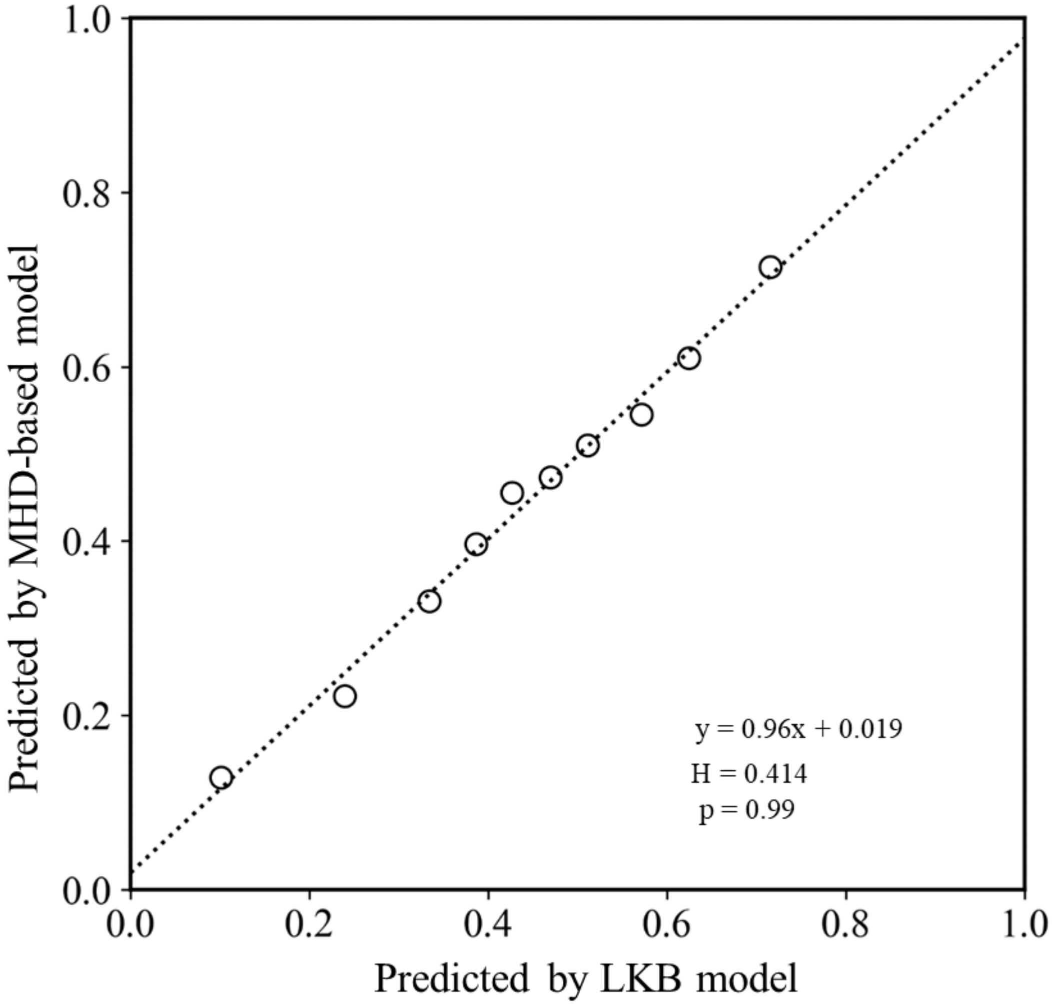

A-PCE: pericardial effusion in any grade; LKB: Lyman–Kutcher–Burman; MHD: mean heart dose

Supplementary Figure 8. Calibration plots using the Hosmer–Lemeshow test of the predicted risk by MHD-based model vs. LKB model for S-PCE

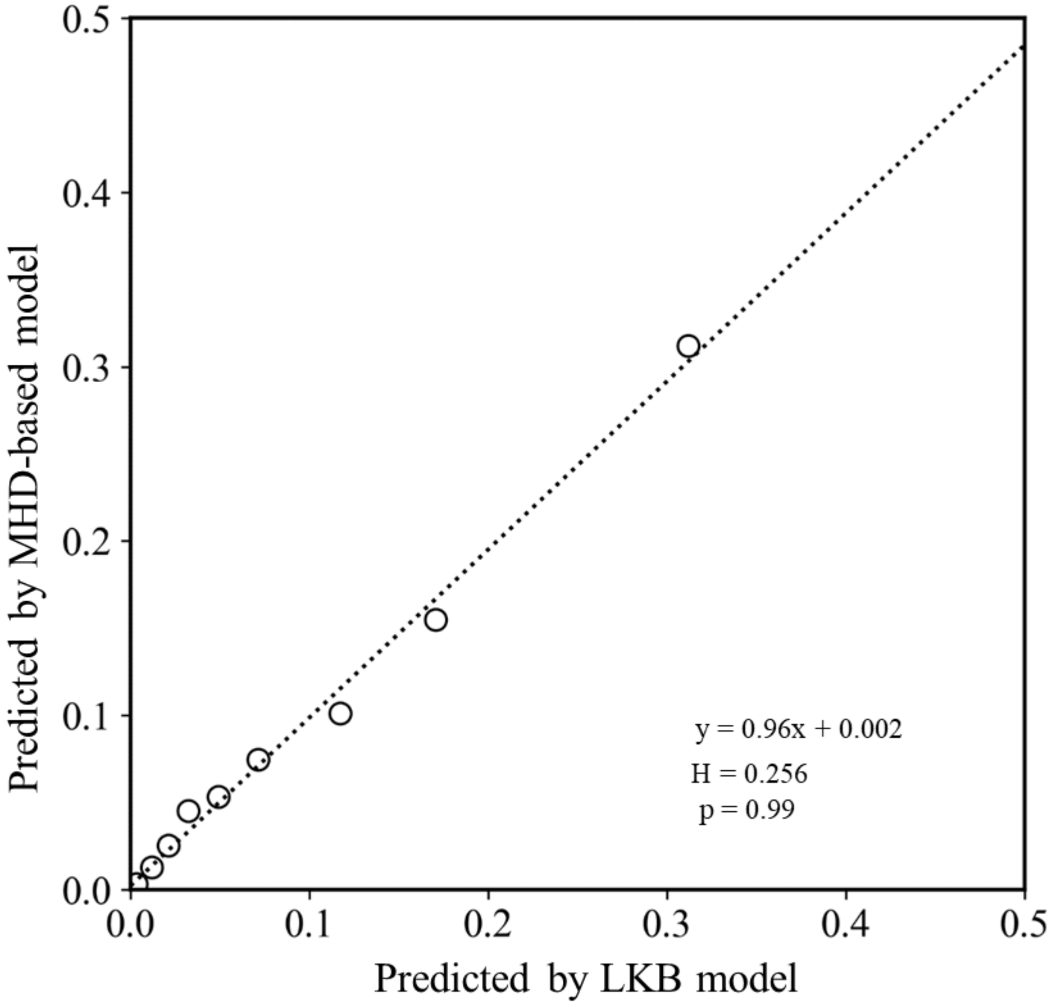

LKB: Lyman–Kutcher–Burman; MHD: mean heart dose; S-PCE: symptomatic pericardial effusion.
